# Slit2N Inhibits Transmission of HIV-1 from Dendritic Cells to T-cells by Modulating Novel Cytoskeletal Elements

**DOI:** 10.1038/srep16833

**Published:** 2015-11-19

**Authors:** Ashutosh Shrivastava, Anil Prasad, Paula M. Kuzontkoski, Jinlong Yu, Jerome E. Groopman

**Affiliations:** 1 Division of Experimental Medicine, Beth Israel Deaconess Medical Center, Harvard Medical School, Boston, Massachusetts, USA; 2 DynaMed, EBSCO Information Services, 10 Estes Street, Ipswich, Massachusetts, USA; 3 Department of Psychiatry, Mclean Hospital, Harvard Medical School, 115 Mill Street, Belmont, Massachusetts, USA.

## Abstract

Dendritic cells are among the first cells to encounter sexually acquired human immunodeficiency virus (HIV-1), in the mucosa, and they can transmit HIV-1 to CD4^+^ T-cells via an infectious synapse. Recent studies reveal that actin-rich membrane extensions establish direct contact between cells at this synapse and facilitate virus transmission. Genesis of these contacts involves signaling through c-Src and Cdc42, which modulate actin polymerization and filopodia formation via the Arp2/3 complex and Diaphanous 2 (Diaph2). We found that Slit2N, a ligand for the Roundabout (Robo) receptors, blocked HIV-1-induced signaling through Arp2/3 and Diaph2, decreased filopodial extensions on dendritic cells, and inhibited cell-to-cell transmission of HIV-1 in a Robo1-dependent manner. Employing proteomic analysis, we identified Flightless-1 as a novel, Robo1-interacting protein. Treatment with shRNAs reduced levels of Flightless-1 and demonstrated its role in efficient cell-to-cell transfer of HIV-1. These results suggest a novel strategy to limit viral infection in the host by targeting the Slit/Robo pathway with modulation of cytoskeletal elements previously unrecognized in HIV-1 transmission.

During sexual contact, HIV-1 enters mucous membranes of the vagina, throat, or rectum^1^ and there, the virus encounters potential target cells, including antigen-presenting cells such as dendritic cells (DCs), which play pivotal roles in innate and adaptive immunity^2^,^3^. DCs are susceptible to infection or internalization of intact virus, and can transmit HIV-1 to CD4^+^ T-cells (hereafter referred to as “T-cells”) with high efficiency^4^. *In vivo,* murine and macaque models of vaginal HIV and SIV transmission, respectively, strongly suggest a central role for DC to T-cell transfer of virus in the mucosa and in secondary lymphoid organs^5^,^6^,^7^,^8^. Moreover, during the acute phase of viral infection, depletion of T-cells by HIV-1 occurs primarily at these mucosal surfaces^9^.

HIV-1 internalized in DCs can be passed to T-cells via an infectious synapse (IS), a complex akin to the immune synapse that forms during MHC Class II antigen presentation^10^. These specialized synapses allow for rapid transmission of the microbe and its shielding from the host immune system^11^. To establish an IS, DC-SIGN, a receptor expressed on DCs, binds to the HIV-1 envelope protein, gp120, and mediates virus internalization and transmission to T-cells^12^,^13^. The role of the actin cytoskeleton during HIV-1 entry, intracellular replication, and transmission via the IS has been well characterized^14^,^15^. At the IS, actin-rich structures such as membrane extensions^16^, filopodia^17^, nanotubes^18^, tunneling nanotubes^19^, and membrane sheets^20^, enhance cell-to-cell transfer of HIV-1 and other retroviruses^15^. Inhibiting cytoskeletal reorganization of HIV-infected DCs reduces cell-to-cell transmission of HIV-1^16^,^17^,^20^.

HIV internalization induces signaling through the tyrosine kinase, c-Src, and the Rho GTPase, Cdc42. Downstream of Cdc42, Wiskott-Aldrich syndrome protein (WASp) is a member of a family of proteins that transduces signals from the cell surface to the cytoskeleton. It binds to and activates the Arp2/3 complex, which modulates the nucleation and polymerization of actin. A different pathway of actin nucleation and elongation depends on formin related protein diaphanous 2 (Diaph2). The Diaph2 dominant pathway leads to the incorporation of newly formed virions into long filopodia originating from productively infected dendritic cells. These actin based elongated structure have been termed viral filopodia^17^.

The Slit proteins are large, secreted, glycoproteins and ligands for the Robo family of receptors^21^. In central nervous system development, Slit-Robo interactions modulate axon guidance and neuronal migration^22^. A role for Slit-Robo signaling also has been demonstrated for tumor angiogenesis, invasion, and metastasis^23^,^24^. In addition, this ligand/receptor pair may inhibit the chemotaxis of various immune cells^25^,^26^,^27^,^28^. *In vivo*, Slit2 is cleaved into a 50-60 kDa C-terminal fragment and a 120-140 kDa N-terminal fragment, Slit2N, which mediates the biological effects of Slit2^29^; therefore, we used Slit2N in our experiments. Slit2N inhibits both X4- and R5-tropic HIV-1 replication in T-cells^30^, as well as HIV-gp120-induced endothelial hyperpermeability^31^ and DC migration^32^. In these studies Slit2N was found to modulate these effects by inhibiting signaling through c-Src and Cdc42^31^,^32^.

These findings prompted us to further address the molecular effects of Slit2N on HIV-1-induced signaling in DCs, with the aim of characterizing how this ligand may alter the cytoskeleton to inhibit virus transmission from DCs to T-cells.

## Results

### Slit2N inhibits the transmission of HIV-1 from DCs to T-cells

**Figure 1:**
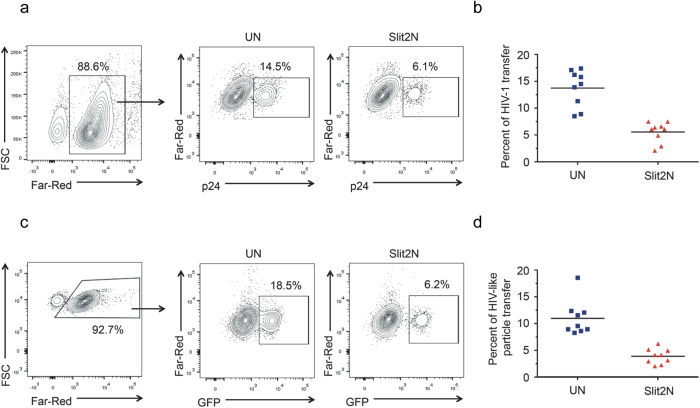
Slit2N inhibits the transfer of HIV-1 and HIV-1-like particles from DCs to T-cells. (**a**) HIV-1 transmission from DCs to T-cells by flow cytometry. DCs were incubated with HIV-1 BaL for 2 h, then washed to remove free virus. Far-Red-labeled T-cells were added, with (“Slit2N”) and without (“UN”) recombinant Slit2N. After 48 h, cells were harvested, and stained with p24-gag antibody (HIV-1 marker), and gated to include only T-cells (left panel). Experiments were carried out in the presence of HIV-1-specific protease inhibitor, indinavir (1 μM). Center and right panels represent the percent of T-cells that have internalized HIV-1. Representative images shown. (**b**) Quantification of HIV-1 transfer as described in (**a**). Data represent the mean ± SEM of 3 independent experiments done in triplicate for untreated T-cells vs. those incubated with Slit2N (p ≤ 0.001, 2-tailed t-test). (**c**) HIV-like particle transfer from DCs to T-cells by flow cytometry. DCs were spinoculated with HIV Gag-iGFP_JRFL virus (HIV-like particles), as described in Methods. Then, Far-Red-labeled T-cells were added, with (“Slit2N”) and without (“UN”) recombinant Slit2N. After 48h, cells were harvested and gated to include only T-cells (left panel). Center and right panels represent the percent of T-cells that have internalized HIV-like particles. Representative images shown. (**d**) Quantification of HIV-like particle transfer as described in (**c**). Data represent the mean ± SEM of 3 experiments done in triplicate for untreated T-cells vs. those incubated with Slit2N (p ≤ 0.001, 2-tailed t-test).

Since transmission of HIV-1 from DCs to T-cells appears to be some 10 to 100 times more efficient than direct infection (4, 5), we initially focused on the effects of Slit2N on cell-to-cell HIV-1 transmission. We used an *in vitro* viral transfer assay that is modeled on early mucosal HIV-1 infection and dissemination in which monocyte-derived DCs take up HIV-1 and transfer the virus *in trans* to T-cells. We maintained the DC to T cell ratio at 1:5, conditions that approximates what is observed in mucosal spaces. To rule out productive infection and replication of HIV-1 in DCs and T-cells, the experiment was carried out in the presence of the HIV-1 protease inhibitor, indinavir. We incubated immature, monocyte-derived dendritic cells (hereafter referred to as “DCs”) with HIV-1 BaL (hereafter referred to as “HIV-1”) for 2 hours, washed the DCs to remove virus that had not been taken up by the cells, and added Far-Red-labeled T-cells, with (Slit2N) and without (UN) recombinant Slit2N. After 48 hours, we used flow cytometry to assess the fraction of Far-Red-labeled T-cells that were also positive for HIV-1 p24. This represented the percent of T-cells to which DCs had transmitted the virus (Fig. 1a). We found a decrease in HIV-1 carriage from DCs to T- cells (~2.6 fold ) if they had been co-cultured with Slit2N than if they had not (Fig. 1b).

As an alternative method to eliminate the contribution of HIV-1 replication in DCs and T-cells, we evaluated the effect of Slit2N on the cell-to-cell transmission of HIV-Gag-iGFP-JRFL, HIV-1 virus-like particles (VLPs) that can undergo only one round of replication^33^. We spinoculated DCs with HIV Gag-iGFP_JRFL VLPs as described in the Methods section, and added Far-Red-labeled T-cells, with (Slit2N) and without (UN) recombinant Slit2N. After 48 hours, we assessed the fraction of Far-Red-labeled T-cells that were also positive for GFP by flow cytometry (Fig. 1c). This represented the percent of T-cells to which DCs had transmitted the VLPs. We found that Slit2N reduced the percent of VLP transfer from DCs to T-cells by approximately 3 fold (Fig. 1d). These data suggest that Slit2N inhibits *in trans* transmission of HIV-1 from DCs to T-cells.

### Slit2N reduces the number of DC membrane extensions and inhibits HIV-1-induced Cdc42 and Src activation

HIV-1 can modulate the cytoskeleton of host cells at multiple points during virus entry, replication, and transmission^15^. Recent examples of cytoskeletal manipulation by HIV-1 during cell-to-cell transmission include formation of various actin-rich membrane extensions, also called filopodia^15^,^18^,^19^. These contacts localize to the IS between infected and uninfected cells and facilitate the transmission of HIV-1, including transmission from DCs to T-cells^16^,^17^.

**Figure 2:**
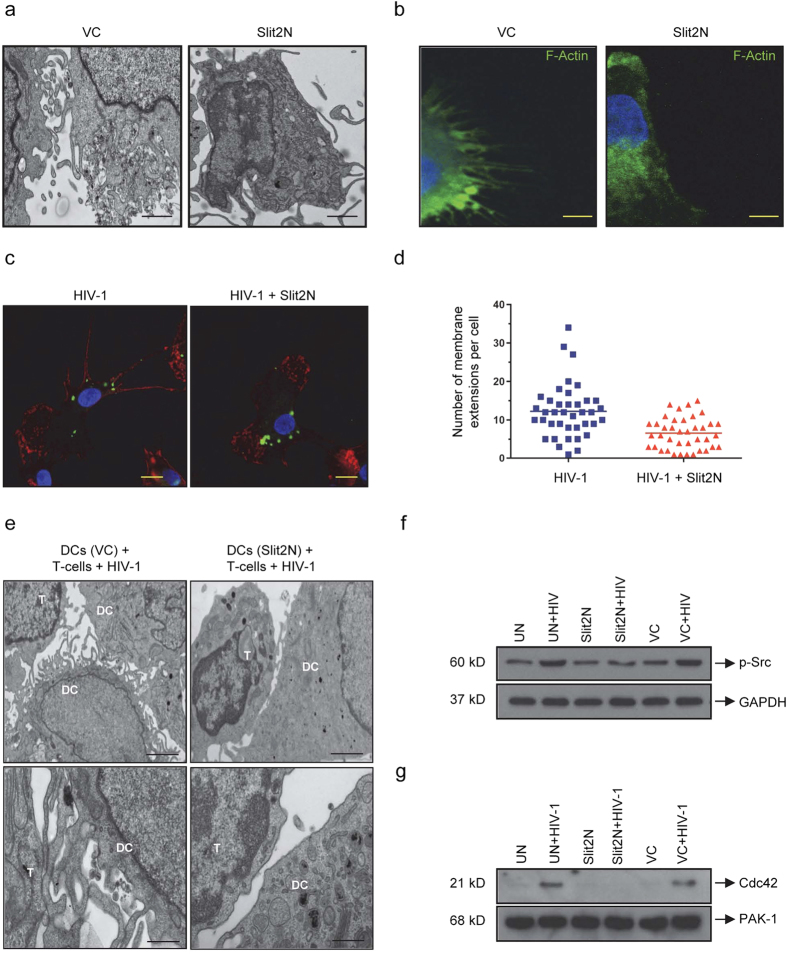
Slit2N reduces DC membrane extension quantity and inhibits HIV-1-induced Cdc42 and Src activation. (**a**) Representative TEM images of DCs, 48h after transduction with Slit2N-expressing adenovirus (Slit2N) or vector control (VC). Scale bars = 500 nm. (**b**) Representative confocal images of DCs transduced as in (**a**). Green: phalloidin-FITC (actin). Scale bars = 5μm. (**c**) Representative confocal images of DCs incubated with HIV-1 BaL for 2 h, then with/without recombinant Slit2N for 24 h. Red: phalloidin (actin); Green: p24 (HIV); Blue: DAPI. Scale bars = 10 μm. (**d**) Average number of membrane extensions/DC, quantitated using Volocity® software. Data represent mean ± SEM of 3 experiments. 40+ cells/experiment per condition (***p = 0.00012, 2-tailed t-test). (**e**) Representative TEM images of contacts between DCs and T-cells. Slit2N-overexpressing DCs (Slit2N) and controls (VC) were incubated with HIV-1 BaL overnight, before adding T-cells. After 72 h, co-cultures were fixed and sectioned. Top images, scale bars = 1000nm; bottom images, scale bars = 200nm. (**f**) Western blot analysis of p-Src in untreated DCs (UN), Slit2N-overexpressing DCs (Slit2N), and controls (VC), with/without overnight incubation with HIV-1 BaL. GAPDH used as loading control. Three experiments performed. (**g**) PAK-1 pull-down and Western blot analysis of activated Cdc42 in untreated DCs (UN), Slit2N-overexpressing DCs (Slit2N), and controls (VC), with/without overnight incubation with HIV-1 BaL. PAK-1 used as loading control. Three experiments performed.

Based on these published studies and our findings (Fig. 1), we hypothesized that Slit2N may inhibit HIV-1 transmission from DCs to T-cells by modulating HIV-1-induced cytoskeletal changes in DCs. To explore this possibility, we examined by transmission electron microscopy (TEM) the morphology of control DCs (VC) and Slit2N-expressing DCs (Slit2N). Control DCs displayed numerous, finger-like, membrane extensions (Fig. 2a, left panel). The morphology of Slit2N DCs was dramatically altered, with significantly fewer extensions relative to controls (Fig. 2a, right panel). We also stained control DCs and Slit2N DCs with phalloidin-actin, captured images by confocal microscopy (Fig. 2b). We observed a notable decrease in the number of membrane extensions/cell in the Slit2N DCs as compared to the controls.

HIV-1 infection can significantly increase the number^16^ and length^17^ of these actin-rich, filamentous structures on DCs. To explore the effect of Slit2N on these HIV-1-induced structures, we incubated DCs with HIV-1 for 2 hours and incubated the cells with and without Slit2N for 24 hours and analyzed images by confocal microscopy (Fig. 2c). We found that Slit2N significantly decreased the number of HIV-1-induced membrane extensions (Fig. 2d). Interestingly, in the DCs incubated with HIV-1 alone, most of the virus localized on or near these extensions, whereas in the DCs incubated with Slit2N, HIV-1 was mostly confined to the cytoplasm (Fig. 2d).

We also examined the effects of Slit2N on DC membrane extensions in the presence of HIV-1 and T-cells. We incubated control DCs (VC) and Slit2N-expressing DCs (Slit2N) with HIV-1 overnight, and T-cells were added next morning. Three days later samples were prepared for TEM. Control DCs displayed many finger-like, membrane extensions, which made numerous contacts with neighboring T-cells (Fig. 2e, “VC” panels); however, Slit2N DCs displayed few membrane extensions and appeared to make little or no contact with proximate T-cells (Fig. 2e, “Slit2N” panels). These data suggest that Slit2N may inhibit the formation of DC membrane extensions and minimize contact between DCs and T-cells, contributing to reduced transmission of HIV-1 from DCs to T-cells at the IS.

Signaling through the c-Src pathway can effect changes to the actin cytoskeleton^34^,^35^. We examined the effects of Slit2N on the activation of key molecules therein, namely c-Src and Cdc42^36^. By Western blot analysis, we examined Src phosphorylation in untreated DCs (UN), in Slit2N-expressing DCs (Slit2N), and in control DCs (VC), with or without HIV-1 (Fig. 2f). While HIV-1 increased the activation of Src in untreated DCs and in vector control DCs, HIV-1 had no effect on its activation in the Slit2N-expressing DCs (Fig. 2f). These data indicate that Slit2N inhibits HIV-1-enhanced activation of Src signaling in DCs.

Since only activated Cdc42 binds to p21 protein-activated kinase 1, (PAK-1)^37^, we examined Cdc42 activation with a PAK-1 pull down assay and Western blot analysis of Cdc42. There was no basal activation in the Slit2N DCs, and incubation with HIV-1 had no effect on Cdc42 activation in these cells (Fig. 2g). There was no basal activation of Cdc42 in untreated DCs or in vector control DCs; however, Cdc42 was activated when these cells were incubated with HIV-1 (Fig. 2g). These data indicate that Slit2N inhibits HIV-1-induced activation of Cdc42 in DCs.

### Slit2N-mediated inhibition of HIV-1 transmission is Robo1-dependent, Robo1 interacts with Flightless-1 (Fli1), and sufficient levels of Fli1 are required for the transmission of HIV-1 from DCs to T-cells

**Figure 3:**
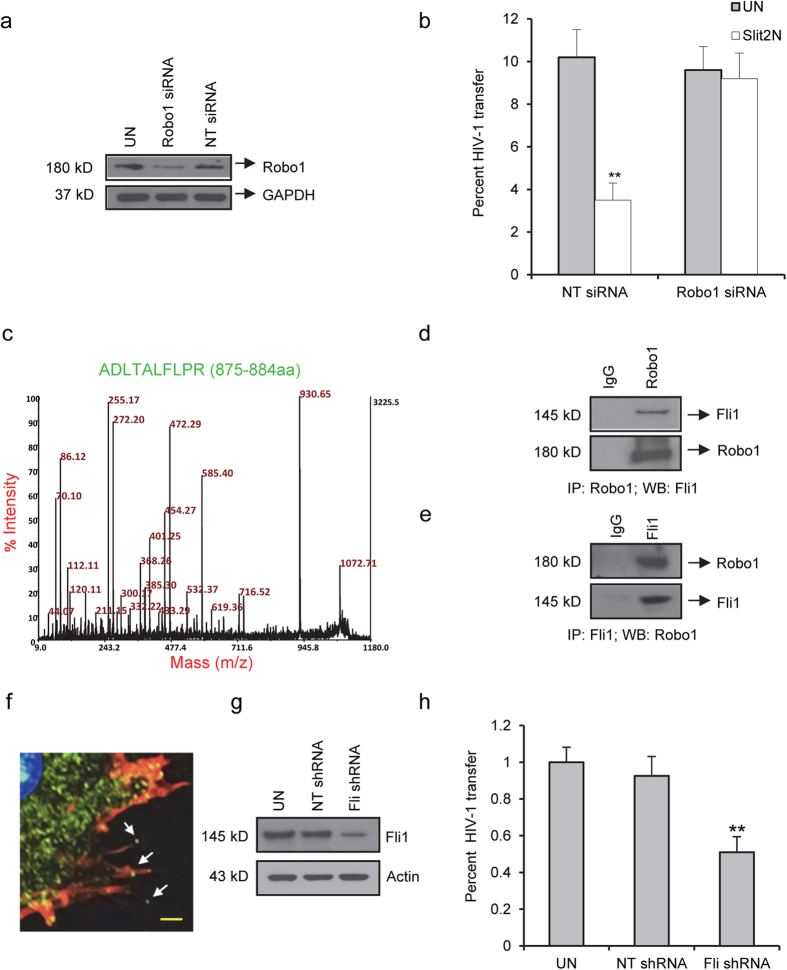
Fli1 interacts with Robo1, and is required for cell-to-cell HIV-1 transmission and its Slit2N-modulated inhibition. (**a**) Western analysis of Robo1 in untreated DCs (UN), and in DCs transduced with non-targeted shRNA lentivirus (NT-shRNA) or Robo1-specific shRNA lentivirus (Robo1-shRNA). GAPDH = loading control. Three experiments performed. (**b**) NT-shRNA DCs and Robo1-shRNA DCs were incubated with HIV-1 overnight, washed, and CellTrace Far-red labelled T-cells were added with/without (“UN”) recombinant Slit2N. The percent of T-cells that have internalized HIV-1 was analyzed by flow cytometry as described in Method section. Data represent mean ± SEM of 3 experiments; (**p = 0.003, 2-tailed t-test). (**c**) DC cultures were lysed and 1 mg of total protein was incubated overnight with 5 μg of Robo1 antibody. The immunoprecipitated material was collected with Protein A-conjugated sepharose beads. The immunoprecipitated material was subsequently run on an SDS-PAGE gel and stained with Coomassie blue. The gel slices were excised and processed to LC/MS/MS to identify peptides that interact with Robo1. Mass spectrometry data for the 10–amino acid representing Flightless1 peptide is shown. (**d**) Robo1 immunoprecipitation/Fli1 Western analysis in DCs. Robo1 = loading control. IgG = antibody control. Four experiments performed. (**e**) Fli1 immunoprecipitation/Robo1 Western analysis in DCs. Fli1 = loading control. Three experiments performed. (**f**) Confocal image of DC. Actin: red; Fli1: green/green dots (see arrows); DAPI: blue. Yellow/orange: colocalization of Fli1 and actin. Scale bar = 5 μm. (**g**) Western analysis of Fli1 in untreated DCs, and in DCs transduced with non-targeted shRNA lentivirus or Fli1-specific shRNA lentivirus. Actin = loading control. (**h**) HIV-1 transmission from DCs to T-cells by flow cytometry. Untreated, NT-shRNA, and Fli-shRNA DCs were incubated with HIV-1, washed, and Far-Red-labeled T-cells and indinavir added. After 48h, T-cell fraction that had taken up HIV-1 was assessed, as previously described. Percent of HIV-1 internalized by T-cells co-cultured with untreated DCs was normalized to “1.” Other percents calculated relative to this control. Data represent mean ± SEM of 3 experiments; (**p ≤ 0.002, 2-tailed t-test).

In earlier studies, we observed that Slit2N-mediated inhibition of HIV-gp120-induced podosome formation and migration of DCs was Robo1-dependent^32^; therefore, we asked if Slit2N-mediated inhibition of HIV-1 transmission from DCs to T-cells was also Robo1-dependent. We transduced DCs with Robo1-specific shRNAs (shRobo1) or with non-targeting shRNAs (NT shRNA), and confirmed reduction of Robo1 expression by Western blot analysis (Fig. 3a). We analyzed the DC to T-cell HIV-1 transfer by utilizing flow cytometry. Recombinant Slit2N significantly decreased the amount of HIV-1 transferred from DCs transduced with non-targeting shRNAs to T-cells; however, it had no significant effect on the HIV-1 transmission to T-cells if experiments were conducted using DCs with reduced levels of Robo1 (Fig. 3b). These data suggest that inhibition of HIV-1 transmission from DCs to T-cells by Slit2N is Robo1-dependent.

Various proteins are known to interact with Robo1 upon its binding with its ligand Slit2, and connect Slit/Robo signaling to actin cytoskeleton reorganization. Although Slit2 can regulate the actin cytoskeleton, detailed knowledge of how Slit-Robo signaling is regulated in immune cells is still lacking. To identify other proteins that may play roles in HIV-1 transmission from DCs to T-cells, we subjected DC lysates to Robo1 immunoprecipitation and mass spectrometry. Fli1 was one of numerous proteins that interacted with Robo1. (Fig. 3c) (complete data set [PASS00320] is available at http://www.peptideatlas.org). Prior studies showed Fli1 to be linked to actin nucleation via formins^38^. Because internalization and trans-infection of HIV-1 are linked to active cytoskeleton reorganization, we sought to further study the role of this protein during HIV-1 transmission.

Fli1, originally characterized in *Drosophila melanogaster*, is a gelsolin-related protein that contains an actin-binding domain and a leucine rich repeat domain^39^,^40^. Through these domains, Fli1 modulates the actin cytoskeletal and signal transduction, respectively^41^. In these capacities, Fli1 is localized in the nucleus, the cytoplasm, and in association with the actin cytoskeleton^42^,^43^,^44^,^45^,^46^. Actin capping is an essential component of cytoskeletal remodeling, in which capping proteins stabilize actin filaments by incorporating onto the growing end of these extensions to arrest further polymerization and to prevent depolymerization^47^. A recent study suggests that Fli1 can also function as an actin capping protein^42^, and Fli1 associates with the actin cytoskeleton, whose remodeling is critical for cell-to-cell transmission of HIV-1; therefore, of the Robo1-interacting proteins identified, we chose to focus on Fli1. We confirmed the interaction in DCs between Robo1 and Fli1 using a Robo1 immunoprecipitation and Fli1 Western blot assay (Fig. 3d), and with a Fli1 immunoprecipitation and Robo1 Western blot assay (Fig. 3e).

We then examined the expression of Fli1 (green), actin (red), and their colocalization (orange/yellow) in DCs by confocal microscopy (Fig. 3f). Fli1 was distributed throughout the cytoplasm. In addition, it colocalized with actin in the filopodia (orange/yellow), and was also expressed near the termini of these projections (green dots highlighted by arrows), which is consistent with its reported roles as an actin binding protein and as an actin capping protein (Fig. 3f). Based on its coimmunoprecipitation with Robo1 and these data, we hypothesized that Fli1 might be required for the efficient transmission of HIV-1 from DCs to T-cells.

To explore this hypothesis, we transduced DCs with a lentivirus that expressed non-targeting shRNAs (NT shRNA) or Fli1-specific shRNAs (Fli shRNA), and confirmed decreased Fli1 expression 24 hours later by Western blot analysis (Fig. 3g). In the presence of the HIV-1 protease inhibitor, indinavir, we incubated DCs with HIV-1 for 2 hours, and subsequently added Far-Red-labeled T-cells. After 48 hours, we used flow cytometry to assess the fraction of Far-Red-labeled T-cells that had taken up HIV-1. We found no significant difference in the levels of HIV-1 internalized by T-cells co-cultured with untreated DCs and of those co-cultured with NT shRNA DCs; however, T-cells that were co-cultured with Fli shRNA DCs internalized approximately half the HIV-1 as T-cells in the other 2 conditions (Fig. 3h). These data indicate that sufficient levels of Fli1 are required for the transmission of HIV-1 from DCs to T-cells.

### Slit2N inhibits HIV-1-induced reduction of Fli1/Robo1 interactions and HIV-1-induced increase in the interaction of actin with Fli1 and Diaph2 in DCs

Formins are a group of proteins that modulate actin polymerization, actin filament formation, and the cytoskeleton^48^,^49^,^50^. Recently, a member of this family, Diaph2, was shown to play a central role in filopodia formation and the transmission of HIV-1 from DCs to T-cells^17^. In addition, another member of the diaphanous family Diaph1 has been shown to induce infectious synapse formation and HIV-1 transmission^16^. Additionally mDia1 can interact with Fli1^38^, an actin-binding protein that complexes with Robo1 (Fig. 3c–e), and for which we observed a role in cell-to-cell HIV-1 transmission (Fig. 3g). Based on these data, we hypothesized that Slit2N may inhibit HIV-1-induced modulation of the DC cytoskeleton by altering associations among Diaph2, Fli1, actin, and Robo1.

**Figure 4:**
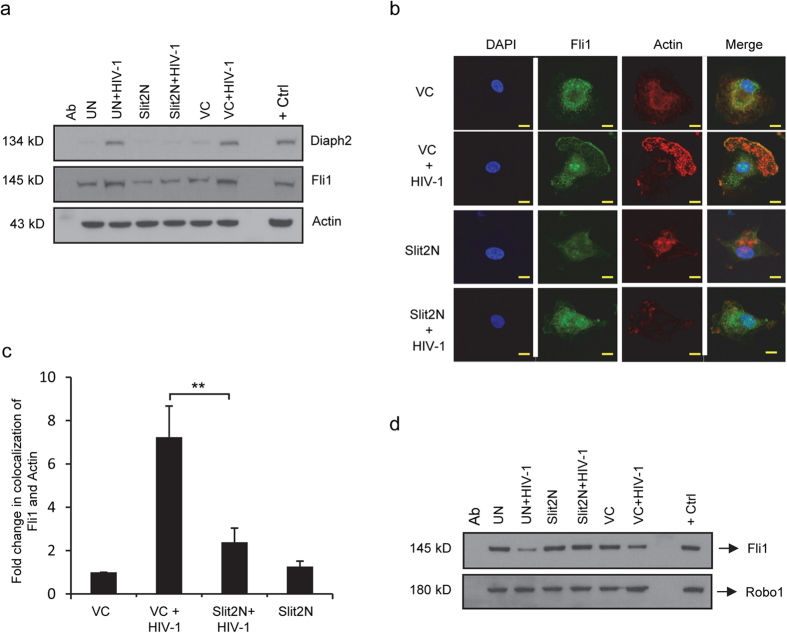
Slit2N inhibits HIV-1-induced colocalization of Actin and Fli1, and the interaction of Actin and Diaph2. (**a**) Actin immunoprecipitation and Western blot analysis of Diaph2 and Fli1 in DCs. Untreated DCs (UN), Slit2N-overexpressing DCs (Slit2N), and control DCs (VC) were incubated with or without HIV-1 BaL overnight, and processed as described in Methods. Actin used as loading control. “Ab” = negative antibody control; “+ Ctrl” = positive control for each protein. Three experiments performed. Representative data displayed. (**b**) Confocal images of the colocalization of Flightless with actin. Scale bars = 10 μm. Three independent experiments performed. Representative images displayed. (**c**) Quantification of the colocalization of Fli1 with actin in DCs, under conditions identical to (**b**), using confocal microscopy and Volocity® software. Data represent the mean ± SEM of 3 independent experiments × 3 randomly chosen cells per condition (**p = 0.00262, 2-tailed t-test). (**d**) Robo1 immunoprecipitation and Fli1 Western blot analysis in untreated DCs (UN), Slit2N-overexpressing DCs (Slit2N) and in control DCs (VC), and incubated with and without HIV-1 BaL overnight, and processed as described in Methods. “Ab” = negative antibody control; “+ Ctrl” = positive control for each protein. Three independent experiments performed. Representative data displayed.

To explore this possibility, we incubated untreated DCs (UN), vector control DCs (VC), or Slit2N DCs (Slit2N), with or without HIV-1 overnight. To assess complexing of actin with Diaph2 and with Fli1, we performed an actin immunoprecipitation, and Western blot analysis of Diaph2 and Fli1. While HIV-1 increased the binding of actin with both Diaph2 and Fli1 in untreated and control DCs, it had no effect on such interactions in the Slit2N DCs (Fig. 4a). We interpret these data to indicate that Slit2N can inhibit HIV-1-enhanced association of actin with Diaph2 and Fli1 in DCs.

To characterize the effects of Slit2N on HIV-1-induced complexing and localization of actin and Fli1, we used the experimental conditions in Fig. 4a, stained control DCs (VC) and Slit2N DCs (Slit2N) with antibodies to Fli1 and actin, and captured images by confocal microscopy. Consistent with our prior data (Fig. 4a), HIV-1 induced a strong colocalization of actin and Fli1 at the leading edge of control DCs (VC); however, even after HIV-1 incubation, in the Slit2N DCs the intracellular distribution of actin and Fli1 remained diffuse, with no discernible increase in colocalization (Fig. 4b–c).

To assess the effects of Slit2N on HIV-1-induced modulation of Robo1 and Fli1 interactions, we performed a Robo1 immunoprecipitation and Western blot analysis of Fli1. HIV-1 decreased the association of Robo1 and Fli1 in untreated (UN) and in control (VC) DCs; however, in the Slit2N DCs, HIV-1 had no effect on the complexing of Robo1 and Fli1 (Fig. 4d). We interpret these data to indicate that in DCs, Slit2N can block the reduced interaction between Robo1 and Fli1, induced by HIV-1.

### Slit2N inhibits the HIV-1-induced complexing of WASp with actin and with Arp2/3 in DCs

**Figure 5:**
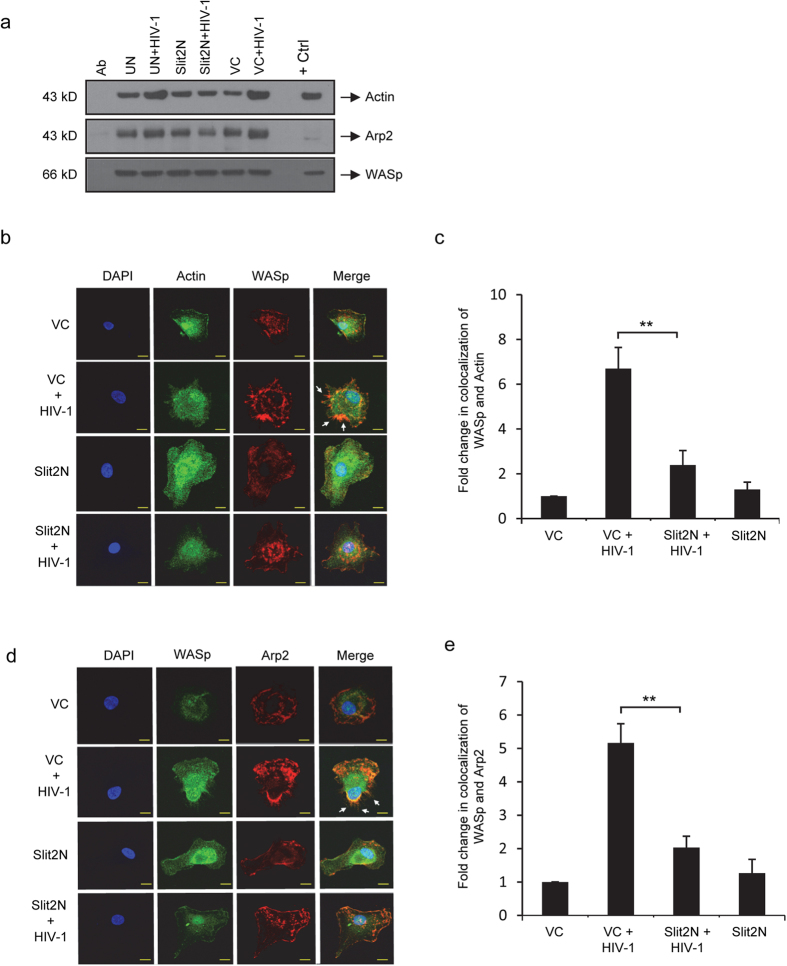
Slit2N inhibits HIV-induced complexing of Actin and Arp2 with WASp in DCs. (**a**) WASp immunoprecipitation and Western blot analyses of actin and Arp2 in untreated DCs (UN), Slit2N-overexpressing DCs (Slit2N), and control DCs (VC), incubated with or without HIV-1 BaL for 1h, and processed as described in Methods. Total WASp used as loading control. (“Ab” = antibody control; “+ Ctrl” = positive control for each protein). Three independent experiments performed. Representative data displayed. (**b**) Confocal images of the colocalization of WASp with actin. Arrows indicate filopodial membrane extensions and scale bars = 10μm. Three independent experiments performed. Representative images displayed. (**c**) Quantification of the colocalization of WASp with actin using confocal microscopy and Volocity® software. Data represent the mean ± SEM of 3 independent experiments × 3 randomly chosen cells per condition (**p = 0.00827, 2-tailed t-test). (**d**) Quantification of the colocalization of WASp with Arp2, in Slit2N-overexpressing DCs (Slit2N) and control DCs (VC), incubated with and without HIV-1 BaL for 1 h. Arrows indicate filopodial membrane extensions and scale bars = 10 μm. Three independent experiments performed. Representative images displayed. (**e**) Quantification and colocalization of WASp with actin using confocal microscopy and Volocity® software. Data represent the mean ± SEM of 3 independent experiments × 3 randomly chosen cells per condition (**p = 0.005375, 2-tailed t-test).

WASp and N-WASp, stimulate actin production by complexing directly with actin, leukocyte-specific protein 1, and Arp2/3. This complexing induces actin nucleation and cytoskeletal rearrangements^51^. We assessed the complexing of N-WASp with actin, and with the Arp2 component of the Arp2/3 complex, with N-WASp immunoprecipitation and Western blot analysis using the same experimental conditions as in Fig. 4; however, since here we evaluated the effects of Slit2N on signaling molecules (WASp and Arp2/3) that are routinely assayed at earlier time points than structural molecules, we incubated DCs with HIV for 1 hour vs. overnight. We found that HIV-1 increased the complexing of both actin and Arp2 with N-WASp in untreated DCs and in vector control DCs (Fig. 5a); however, HIV-1 had no effect on the complexing of these proteins in the Slit2N DCs (Fig. 5a). These data indicate that Slit2N inhibits HIV-1-induced complexing of N-WASp with actin and with the Arp2/3 complex in DCs; therefore, Slit2N may block HIV-1-induced actin nucleation and rearrangement of the cytoskeleton in DCs.

To assess the effects of Slit2N on the HIV-1-induced cellular localization of these proteins in vector control DCs and in Slit2N DCs, we incubated the cells with or without HIV-1 for 1 hour, stained the cells and analyzed by confocal microscopy (Fig. 5b). Consistent with Fig. 5a, HIV-1 induced a significant increase in colocalization of WASp and actin in control DCs (Fig. 5b,c). Moreover, their coexpression was localized to filopodial, membrane extensions (Fig. 5b, arrows), which are morphologically consistent with control DC membrane extensions induced by incubation with HIV-1 and T-cells (Fig. 2d, left panels). Actin and WASp were diffusely expressed in Slit2N DCs (Fig. 5b). Even after incubation with HIV-1, very little colocalization was detected (Fig. 5b,c), and no discreet filopodia extended from the DC cell membrane (Fig. 5b). This is consistent with the morphology of Slit2N DCs, incubated with HIV-1 and T-cells (Fig. 2d, right panels).

We also visualized the expression of WASp (green), Arp2 (red), and the colocalization of WASp and Arp2 (yellow/orange), under the same experimental conditions. Consistent with Fig. 5a, HIV-1 induced a significant colocalization of WASp and Arp2 in control DCs (Figs 5d,e). Again, their coexpression was localized to filopodial, membrane extensions (Fig. 5d), similar to those formed by control DCs after incubation with HIV-1 and T-cells (Fig. 2d, left panels). HIV-1 induced very little colocalization of WASp and Arp2 in the Slit2N DCs (Fig. 5d,e). Likewise, HIV-1 did not induce the formation of DC membrane extensions (Fig. 5d).

These data indicate that HIV-1 modulates reorganization of the DC cytoskeleton, and promotes the complexing of WASp with actin and with the Arp2/3 complex in filopodial extensions of the DC cell membrane. Moreover, Slit2N appears to inhibit HIV-1-induced complexing of WASp, actin, and Arp2/3, and to inhibit the formation of DC membrane extensions, and their colocalization therein.

## Discussion

AIDS arises when HIV-1 kills sufficient numbers of helper T-cells to compromise the immune system. HIV-1 can directly infect these T-cells; however, transmission from DCs to T-cells is far more efficient. We evaluated the effects of Slit2N on the transfer of HIV-1 from DCs to T-cells. Recent studies showed that Slit2N inhibits HIV-1 transmission to T-cells and its replication therein^30^,^52^, and we now observe how Slit2N can inhibit DC to T-cell transmission, a mode of transfer that utilizes unique cytoskeletal components.

Slit2N is a glycoprotein that is constitutively expressed in neuronal cells and known to regulate the directional migration of various cell types^26^,^53^. Equilibrium binding experiments suggest that the dissociation constants for the interactions of Robo1 with hSlit2 are in the ~4–5 nM range^54^. This nM concentration range of Slit2 has been shown to inhibit migration of Langerhans cells^27^. In the present study, we used recombinant Slit2 at the concentrations of 1000 ng/ml, equivalent to 8.16 nM and within a physiological range.

To gain entry into target cells, HIV-1 uses the CD4 receptor and a coreceptor. HIV-1 variants that use CCR5 as an entry coreceptor are termed R5 and those that use CXCR4 are termed X4. HIV-1 present in infected individuals frequently comprises a mixture of R5 and X4, however, virus isolated from individuals newly infected through sexual or mother-to-child transmission is predominantly R5^55^,^56^. There is a yet-unknown selective mechanism that favors R5 virus during transmission and/or the early phases of infection in the host^57^. Because we focused on the effect of Slit2 on early stage HIV-1 transmission of DC to T cells that takes place in mucosal spaces, we used an R5 strain of HIV-1 in this study.

HIV-1 can activate Cdc42 that in turn induces formation of actin based membrane extensions to facilitate cell-to-cell HIV-1 transmission^16^. Silencing of Cdc42 using siRNA as well as its specific inhibition using Secramine A, both inhibit actin based membrane extensions and decrease HIV-1 transfer at the infectious synapse^16^. HIV-1 can increase the number of membrane extensions on DCs, while HIV-Δenv (gp120-deleted HIV-1) fails to do so^16^. Also, gp120 alone can increase the number of membrane extensions on DCs^16^. We also incubated DCs with HIV Gag-iGFPΔEnv and did not observe an increase in the number of membrane extensions as determined by F-actin immunostaining and confocal microscopy (data not shown). These results suggest that the engagement of HIV-1 to the DC-SIGN receptor is important in this process.

The binding of the HIV-1 envelope protein, HIV-1^env^, to DC-SIGN initiates signaling through the c-Src/Cdc42/WASp/Arp2/3 pathway in DCs^16^. This is one of two major pathways that modulates actin nucleation, formation of membrane extensions, and cell-to-cell transfer of HIV-1^16^. The other major Cdc42 effectors that modulate these functions are the formins, Diaph1^16^ and Diaph2^17^,^58^,^59^. We observed that HIV-1 enhanced the association of WASp with actin and Arp2/3. We also observed that HIV-1 increased the binding of Diaph2 with Fli1 and actin. Diaph2 is known to be involved in the formation of viral filopodia in an HIV-Env independent manner^17^. Although we also found involvement of Diaph2 in the formation of membrane extension, the membrane structures that we studied were dependent on HIV-Env, therefore much closer to what Nikolic *et al.* have observed in their studies^16^.

Our studies indicate that the inhibitory effects of Slit2N on HIV-1 transmission from DCs to T-cells are Robo1-dependent. This is consistent with Robo1 mediating Slit2N inhibition of HIV-1-gp120-enhanced DC migration and podosome formation^32^. In these experiments, the binding of Slit2N to Robo1 appeared to alter the interaction between Robo1 and leukocyte-specific protein 1, a novel member of the WASp/Arp2/3/actin complex that affects the migration of immature DCs^32^. In an effort to characterize novel DC proteins that complex with Robo1, and might influence cell-to-cell transmission of HIV-1, we identified Fli1.

Fli1 is a member of the gelsolin family of proteins that function as actin-binding proteins and as coactivators for nuclear hormone receptor-mediated transcription. These proteins modulate actin cytoskeletal remodeling and hormone-induced intracellular signaling^43^,^44^. Fli1 can bind to both globular and filamentous actin, and enhances transcription of various hormone receptor-mediated genes^39^,^43^; thus, it can be associated with the cytoskeleton or localized to the nucleus^44^.

**Figure 6:**
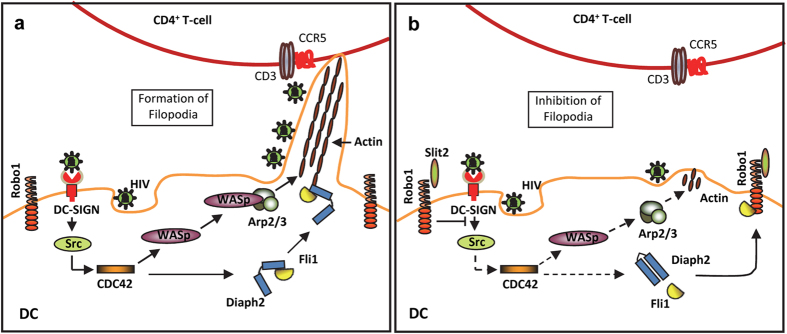
Proposed model of Slit2/Robo1 effects on HIV-1-induced filopodia formation and DC-to-T-cell transmission of HIV. (**a**) HIV-1 induces actin nucleation, actin filament stabilization, and filopodia formation, which enhance the transmission of the virus from DCs to T-cells. (**b**) Slit2 via Robo1 inhibits HIV-1-induced actin nucleation, actin filament stabilization, filopodia formation, and transmission of the virus from DCs to T-cells.

We found that sufficient levels of Fli1 were required for efficient transfer of HIV-1 from DCs to T-cells. This led us to evaluate the effects of HIV-1 and Slit2N on the complexing and localization of Fli1 with actin and with Robo1 in DCs. HIV-1 reduced the interaction between Fli1 and Robo1, and enhanced the interaction between Fli1 and actin. As discussed above, HIV-1 also enhanced the interaction of Diaph2 and actin, and Slit2N inhibited these virus-induced effects. These findings are consistent with a model proposed by Higashi *et al.*^38^ in which activated Rho GTPases release a formin mDia1 from its autoinhibitory conformation and Fli1 binds to its C-terminus, enhancing actin assembly and the formation of membrane extensions. Based on this and our data, we propose the following model for the effects of HIV-1 and Slit2N on the formation of DC filopodia and the transfer of HIV-1 from DCs to T-cells at the IS (Fig. 6).

The interaction of HIV-1 with DC-SIGN on the surface of DCs initiates signaling through Src and Cdc42. WASp/Arp2/3 and Diaph2, the two independent pathways, both downstream of Cdec42 and initiate actin nucleation and formation of membrane extensions. Upon activation, WASp complexes with Arp2/3, and Diaph2 complexes with Fli1. Both the WASp/Arp2/3 complex and the Diaph2/Fli1 complex initiate actin polymerization, and Fli1 stabilizes the growing actin filaments by functioning as a capping protein. These protein interactions induce the formation of filopodial membrane extensions on DCs, which significantly enhance the transmission of HIV-1 from DCs to T-cells (Fig. 6a). When Slit2 binds to Robo1 on the surface of DCs, HIV-1-induced signaling through Src/Cdc42 and to its downstream effectors is inhibited. This leads to destabilization of existing actin filaments in the filopodia, and the number and size of filopodia are significantly reduced (Fig. 6b). Inhibiting the formation and stabilization of DC membrane extensions appear to be the mechanisms by which Slit2 and Robo1 inhibit the transmission of HIV-1 from DCs to T-cells.

We found that Slit2N via Robo1 inhibited the formation and compromised the stability of filopodia on DCs by blocking HIV-1-induced signaling through both WASp/Arp2/3 and Diaph2/Fli. This significantly reduced transmission of virus from DCs to T-cells at the IS. It is hypothesized that for sexually acquired HIV-1, mucosal DCs internalize the virus and transmit it to T-cells in the mucosa, or they may migrate to lymph nodes and infect an even larger pool of target T-cells; therefore, these findings suggest that Slit2N/Robo1 may prevent transmission of HIV-1 from DCs to T-cells in the mucosa of the mouth, vagina or rectum, and in the lymphatics. Our results support therapeutically exploiting Slit2 and Robo1 to limit cell-to-cell transmission of HIV in the host, and highlight a novel role for Fli1 in HIV-1 transmission.

## Methods

### Cells, HIV-1 and constructs

Buffy coats were obtained from the Blood Transfusion Service, Massachusetts General Hospital, Boston, MA, in compliance with the Beth Israel Deaconess Medical Center Committee on Clinical Investigations (CCI) protocol #2008-P-000418/5. Buffy coats were provided at this institution for research purposes; therefore, no informed consent was further needed. In addition, buffy coats were provided without identifiers. This study was approved by Beth Israel Deaconess Medical Center’s CCI, Institutional Review Board, and Privacy Board appointed to review research involving human subjects. The experimental procedures were carried out in strict accordance with approved guidelines.

Peripheral blood mononuclear cells (PBMCs) were isolated from buffy coat by centrifugation, using a Ficoll-Paque density gradient (GE Healthcare Biosciences, Piscataway, NJ). CD14^+^ cells were isolated using a monocyte-positive selection kit per manufacturer’s protocol (STEMCELL Technologies, Inc., Vancouver, BC). To generate iMDDCs, the monocytes were cultured in RPMI supplemented with 10% FCS, 2 mM L-glutamine, 100 IU/ml penicillin, 100 μg/ml streptomycin, 1% nonessential amino acids, 1 mM sodium pyruvate, 500 IU/ml GM-CSF (PeproTech Inc., Rocky Hill, NJ), and 500 IU/ml IL-4 (PeproTech Inc.). After 5 to 7 days, iMDDCs were characterized by evaluating the expression of DC-SIGN and CD1a with appropriate antibodies using FACS analysis.

Autologous T-cells were isolated from PBMCs using a negative selection kit per manufacturer’s protocol (STEMCELL Technologies, Inc.). These cells were activated with PHA-L (1 μg/ml) and maintained in complete culture medium supplemented with IL-2 (30 U/ml) (Advanced Biotechnologies, Inc., Columbia, MD) at 2 × 10^6^ cells/ml. Purity of these T-cells was analyzed using CD3 and CD4 staining and flow cytometry.

HIV-1 BaL was obtained from the NIH AIDS Research and Reference Reagent Program, National Institute of Allergy and Infectious Disease, NIH. To prepare HIV-1 stocks, PBMC derived T-cells were cultured with HIV-1 BaL for 7 d. Fresh T-cells, suspended at 1 × 10^6^ cells/ml were added at day 7. At day 14 after initial viral inoculation, the supernatant was harvested and stored at −80 °C. p24 viral antigen in the supernatants was quantified by ELISA (Perkin Elmer Life and Analytical Sciences, Shelton, CT).

To construct the Slit2N ENTRY clone, the Slit2N gene was amplified from PCMV-ENTRY-Slit2N (OriGene Technologies, Inc., Rockville, MD) using the following primers: 5′- CACCATGCGCGGCGTTGGCTGGCAGATGC -3′ and 5′- GGGACCATGGGTG GAGAAAACTC -3′. The PCR product was cloned into PENTR™/D-TOPO® vector. A Slit2N expression clone with a C-terminal V5 tag was generated by performing the LR reaction between pENTR/D-TOPO-Slit2N and PAD/CMV/V5-DEST (Life Technologies Corp., Carlsbad, CA). After cutting with PAC1, the expression construct was transfected into HEK-293A cells (ATCC, Manassas, VA) to generate the adenoviral stock. The Slit2N adenovirus was transduced into human iMDDCs using ViraPower™ adenoviral expression system (Life Technologies Corp.) per manufacturer’s protocol, and Slit2N expression was confirmed by Western blot analysis 24 to 48 h later using an antibody specific to Slit2.

### Antibodies and reagents

All reagents used in this study were molecular grade and procured from Sigma-Aldrich Co. (St. Louis, MO), unless otherwise indicated. Recombinant Slit2N was from Peprotech (Rocky Hill, NJ). The Slit2 (ab7665) and Robo1 (ab85312) antibodies were obtained from Abcam, Inc. Phosphorylated c-Src (2466) and Cdc42 (6943) antibodies were obtained from Cell Signaling Technology (Danvers, MA). Actin (SC8432), Arp2 (SC137250), WASp (SC365859), Fli1 (SC30046, SC21716), Dia2 (SC55539), GAPDH (SC25778) and FITC-conjugated actin (SC1615FITC) antibodies were obtained from Santa Cruz Biotechnology, Inc. (Santa Cruz, CA). Rhodamine-Phalloidin (R415) and CellTrace Far-Red DDAO-SE (C34553) were obtained from Life Technologies Corp. FITC-conjugated p24 GAG (6604665) antibody was obtained from Beckman Coulter, Inc. (Brea, CA). DC-SIGN and CD1a antibodies were obtained from R&D Systems, Inc. (Minneapolis, MN).

### Assessing HIV-1 transfer by flow cytometry

To analyze the effect of Slit2N on HIV-1 transfer from DCs to T-cells *in trans*, experiments were carried out in the presence of HIV-1-specific protease inhibitor, indinavir (1 μM). DCs (2.0 × 10^5^) were incubated with HIV-1 BaL [200 ng/ml p24 gag] for 2 hours, washed thrice in 1 × PBS to remove untrapped virions, and replated. Subsequently, CellTrace Far-Red-labeled T-cells (1.0 × 10^6^) were added, with and without recombinant Slit2N [1000 ng/ml] (Peprotech, Rocky Hill, NJ). After indicated times, cells were stained for p24 (HIV-1 marker) and analyzed by flow cytometry. Far-Red-labeled T-cells were analyzed for p24 co-expression by FACS analysis, from a total of 10,000 acquired events.

R5-tropic HIV Gag-iGFP_JRFL was obtained from Dr. Benjamin Chen (Mount Sinai Medical School, NY, USA) and described herein^33^. For the production of fluorescent VLPs, the plasmid was transiently transfected into 293T cells using Lipofectamine 2000 (Invitrogen). Supernatants were harvested 2 days post-transfection. For VLP purification, pooled supernatants were filtered (0.45 μm), and pelleted through a 20% sucrose cushion (100,000 × g, for 90 min at 4 °C). VLPs were titrated by p24 ELISA.

To establish single round infectivity assay, 2 × 10^5^ DCs/well were spinoculated in flat bottom 96-well microtiter plates, incubated with 100 μl of VLP stock (100 ng of p24/ml), 1000 rpm for 1 hour^60^. The cells were then co-cultured with Far-Red-labeled T-cells in the presence or absence of Slit2N for the next 48 hours. Flow cytometry analysis performed as described above.

### Western blotting and immunoprecipitation

iMDDCs were left untreated, or transduced with a Slit2N-expressing adenovirus (Slit2N) or a control adenovirus (VC). After 24 h (or time indicated), HIV-1 BaL ([200 ng/ml] p24 gag) was added to the DC culture (if notated). After indicated incubation time, samples were collected in RIPA buffer. Protein lysates were separated on NuPAGE precast gels (Life Technologies Corp.), transferred to 0.45 μm nitrocellulose membranes (Bio-Rad Laboratories, Hercules, CA), and probed with appropriate primary antibodies followed by incubation with their respective secondary antibodies. Proteins were visualized with Western Lightning Plus ECL Substrate (PerkinElmer, Waltham, MA).

For immunoprecipitation assay, DCs were left untreated or transduced with a control adenovirus (VC) or a Slit2N-expressing adenovirus (Slit2N). After 24 h, HIV-1 BaL ([200 ng/ml] p24 gag) was added to the DC cultures (as indicated), and incubated for times indicated. DCs were lysed with cell lysis buffer (Cell Signaling Technology). Immunoprecipitation was performed as previously described^32^.

### shRNA knockdown

To reduce Robo1 and Flightless-1 expression in iMDDCs, we used Flightless-1 and Robo1-specific shRNA lentiviruses and non-targeting shRNA lentivirus as per manufacturer’s protocol (Santa Cruz Biotechnology, Inc., Dallas, TX). Dendritic cells were infected with respective shRNA containing lentivirus at a MOI of 20 and plates were allowed to incubate overnight. Next morning, the culture medium was replaced with 1 ml of complete medium. After 24 h, cell lysates were assessed by Western blot analysis for the expression of the target proteins.

### Cdc42 activation assay

Cdc42 activation was assessed with a Cdc42 activation assay kit (EMD Millipore Corp, Billerica, MA) per manufacturer’s protocol.

### Confocal microscopy

Intracellular localization and colocalization of proteins were evaluated by confocal microscopy as described earlier^32^. Stained cells were analyzed using a Zeiss Axioimager Z1 upright microscope (Carl Zeiss, Jena, Germany). Fluorophores were visualized using the following filter sets: 405 nm excitation for DAPI; 488 nm excitation and band pass 505–530 emission filter for Alexa 488 and FITC; 543 nm excitation and band pass 560–615 for Alexa 543. Images were taken using 40x Zeiss Apochromat Oil, 1.3 NA, DIC (UV) VIS IR or by using 63X Zeiss Apochromat Oil, 1.4 NA, DIC, IR a 40x numerical aperture 1.4 oil immersion objective lens and 2x optical zoom (Carl Zeiss, Jena, Germany). Figures were made using Adobe Photoshop CS4 software (Adobe Systems, San Jose, CA). The colocalization of Fli1 with actin, WASp with actin, and WASp with Arp2 in DCs was assessed using confocal microscopy and Volocity® software (PerkinElmer, Waltham, MA). Briefly, the Manders (M) coefficient, which represents the percentage of pixels in the red channel which intersect with signal in the green channel, was set to “1” in vector control (VC)-treated DCs. Fold change in protein colocalization under all other conditions was calculated relative to this control.

### Transmission electron microscopy

Cells were fixed in 2% formaldehyde and 1.5% glutaraldehyde in 0.2 M sodium phosphate buffer (pH 7.4) at room temperature for 2 h. Cells were washed in sodium phosphate buffer and postfixed in 2% osmium tetroxide for 2 h. Cells were dehydrated and then embedded in epon. After ultramicrotome sectioning, sections were analyzed using a JEOL 1200EX electron microscope (JEOL USA Inc., Peabody, MA).

### Liquid chromatography tandem mass spectrometry (LC/MS/MS)

LC/MS/MS was used to identify peptides that interact with Robo1. DC cultures were lysed in cell lysis buffer and 1 mg total protein was incubated overnight with 5 μg of Robo1 antibody. The antigen-antibody complexes were immunoprecipitated with Protein A-conjugated sepharose beads. The immunoprecipitated material was subsequently run on an SDS-PAGE gel. SDS-PAGE gel slices were processed for LC/MS/MS-based protein identification on reduced, alkylated, trypsin-digested peptide samples, prepared according to standard MS protocols. Peptides were identified by MALDI-TOF/TOF (MALDI-time of flight/time of flight) mass spectrometry on an ABSciex 4800 Plus instrument. Protein Pilot 3.0 software (ABSciex) was used for peptide and protein identification. Complete data set (PASS00320) is available at http://www.peptideatlas.org.

### Statistics

Differences between groups were calculated using a standard 2-tailed Student’s t-test. p-values ≤ 0.05 were considered statistically significant.

## Additional Information

**How to cite this article**: Shrivastava, A. *et al.* Slit2N Inhibits Transmission of HIV-1 from Dendritic Cells to T-cells by Modulating Novel Cytoskeletal Elements. *Sci. Rep.* **5**, 16833; doi: 10.1038/srep16833 (2015).
